# Transitioning Amiodarone Tablet Manufacturing: A Comparative Study of Batch and Continuous Wet Granulation

**DOI:** 10.3390/ph19060850

**Published:** 2026-05-29

**Authors:** Ju-Hyun Yoon, Chae-Won Jeon, Joo-Eun Kim

**Affiliations:** 1 Department of Biopharmaceutical Chemistry, Kookmin University, Seoul 02707, Republic of Korea; wnguss2@naver.com (J.-H.Y.); great2k1@naver.com (C.-W.J.); 2 Biopharmaceutical Chemistry Major, School of Applied Chemistry, Kookmin University, Seoul 02707, Republic of Korea; 3 Department of Pharmaceutical Engineering, Kookmin University, Seoul 02707, Republic of Korea

**Keywords:** amiodarone hydrochloride, twin-screw granulation, design space, process optimization

## Abstract

**Background/Objectives**: The objective of this study was to design and optimize a continuous wet granulation process for Amiodarone hydrochloride tablets using a Design of Experiments approach. The study compared and evaluated the characteristics of granules and tablets produced via a high-shear mixer (batch process) and a twin-screw granulator (continuous process). **Methods**: For process optimization, a central composite design was applied to establish a design space, defining screw speed and milling size as critical process parameters (*X*) and dissolution rate, flowability, assay, disintegration time, and friability as dependent variables (*Y*). **Results**: Comparative results between the two processes revealed no significant differences in in-process control parameters, and all formulations successfully met the target dissolution profiles. Notably, the similarity factor (*f*_2_) was calculated to be above 50, through which dissolution equivalence was successfully demonstrated with a high level of statistical certainty. Regarding process efficiency, lead time measurements confirmed that the continuous process dramatically reduced manufacturing time by more than 80% compared to the batch process. **Conclusions**: This study validates the feasibility of converting batch-based drug manufacturing to a continuous platform without altering the formulation, presenting an effective process strategy for enhancing productivity and operational efficiency in the pharmaceutical industry.

## 1. Introduction

Recently, the pharmaceutical industry has been accelerating a paradigm shift from traditional batch manufacturing toward continuous manufacturing (CM) systems to simultaneously maximize production efficiency and ensure stringent quality control [1]. CM represents an innovative production methodology in which all stages—from raw material input to the final finished product—are executed within a seamless, integrated flow, emerging as a next-generation standard in the industry [2,3]. Unlike batch processes, where unit operations are conducted independently—often resulting in substantial time and resource expenditures due to inter-process lag times, material handling, and offline quality inspections—CM enables a dramatic reduction in production lead times and minimizes the risks of quality variability through the implementation of real-time monitoring and feedback control systems [4,5,6].

The cornerstone of a CM system hinges on the precise identification and control of critical Process Parameters (CPPs). By seamlessly integrating with process analytical technology (PAT), CM systems facilitate the real-time monitoring of critical material attributes (CMAs) and critical quality attributes (CQAs). This capability ensures product uniformity and process robustness by allowing for the real-time adjustment of CPPs in response to subtle in-process variations [7,8,9,10].

Furthermore, this technological framework paves the way for rapid mass production and flexible throughput adjustment in response to emergencies, such as supply chain disruptions or global pandemics. Ultimately, it enhances product reliability by minimizing human error and ensuring consistent quality throughout the production lifecycle [11,12,13].

In particular, since the transition of the wet granulation process profoundly influences the physical characteristics of granules and the subsequent drug dissolution behavior, a comprehensive understanding of the correlation between the batch-based high shear mixer (HSM) and the continuous twin screw granulation (TSG) is imperative.

Within the TSG-based continuous granulation process, various parameters such as screw configuration, liquid-to-solid (L/S) ratio, and feed rate must be considered. Among these, the twin-screw speed can serve as a dominant CPP depending on the formulation attributes, as it directly dictates the mechanical shear force and residence time distribution (RTD) of the granules [14,15,16,17].

Although the L/S ratio is highly critical as a dominant CPP in TSG, it was kept constant in this study to prevent any composition changes and preserve the validated batch formulation matrix. Under this constraint, subtle fluctuations in screw speed significantly affect granule density, particle size distribution (PSD), and tablet hardness. Therefore, establishing an optimized design space (DS) for this specific variable is a prerequisite for a successful transition to a continuous manufacturing system [18,19].

The primary objective of this study is to transition the established batch-based manufacturing process for Amiodarone hydrochloride (HCl) tablets to a CM system and comparatively evaluate the quality equivalence between the two processes. To this end, the physicochemical properties of granules and tablets manufactured by HSM and TSG were analyzed from multiple perspectives.

Specifically, this research aimed to systematically investigate the influence of twin-screw rotation speed, which was identified as a critical process parameter tailored to this specific formulation and a key success factor for the continuous process. To derive the interactions between process variables and establish optimal conditions, a Design of Experiment (DoE) approach based on central composite design (CCD) was implemented. Through these methodologies, the study seeks to validate the feasibility of the continuous manufacturing process and its potential to enhance production efficiency within the derived DS.

## 2. Results and Discussion

To systematically evaluate the success of transitioning from the traditional batch platform to the CM platform, a clear quality evaluation framework was established. The evaluated pharmaceutical parameters were classified into ‘Mandatory’ and ‘Informative’ attributes based on compendial standards and process monitoring significance (Table 1). Mandatory attributes serve as critical quality parameters required for regulatory release, whereas informative attributes function as process performance indicators.

### 2.1. Comparative Assessment of Granules

#### 2.1.1. Comparative Analysis of Granule Flowability

In this study, the flowability of batch and CM granules, milled using screen sizes of 1.0, 2.0, and 3.0 mm, was precisely evaluated in accordance with the United States Pharmacopeia (USP) <1174> Powder Flow method [20]. Granules produced via the batch process exhibited a bulk density of 0.6500 g/mL and a tapped density of 0.7200 g/mL. The resulting Carr’s index (CI) was 9.7%, demonstrating ‘Excellent’ flowability according to USP standards. In contrast, for the granules produced by the continuous manufacturing process, no significant correlation was observed between flowability and variations in twin-screw speed or milling size. However, the CI for these granules ranged from 20% to 30% on average, indicating reduced flowability compared to those from the batch process. (Table 2).

#### 2.1.2. Comparative Analysis of Granule Content Uniformity

In this study, the content uniformity of amiodarone HCl within granules manufactured via HSM and TSG processes was evaluated. Quantitative analysis of the drug content (assay) for each process using high-performance liquid chromatography (HPLC) revealed that the mean assay of granules produced by the conventional batch HSM process was 96.8 ± 1.1%, while those produced by the continuous TSG process showed a mean of 94.2 ± 1.3%. Both processes successfully ensured stable drug content within the defined specifications (Table 3). Notably, statistical comparison of the mean assays and standard deviations between the two manufacturing methods showed no significant differences in terms of content uniformity. This indicates that homogeneous mixing between the active ingredient and excipients and effective granule formation were achieved even within the short residence time of the TSG process. Consequently, these findings provide a critical scientific basis demonstrating that the continuous manufacturing process can offer a level of process reproducibility and quality consistency equivalent to that of the conventional batch process.

#### 2.1.3. Comparative Analysis of Milled Granule Particle Size Distribution

**Figure 1:**
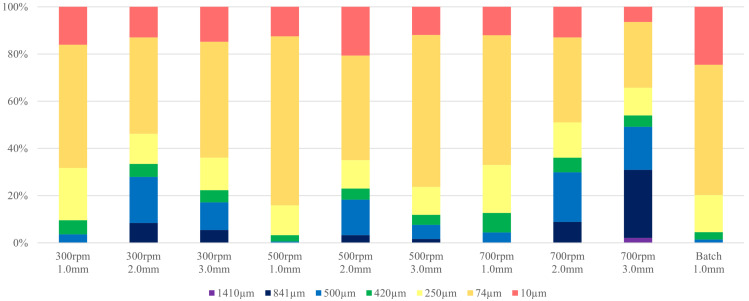
Particle size distribution graph: batch vs. continuous process for milled granules.

To investigate the impact of different manufacturing processes on the particle size characteristics of granules, a comparative analysis of PSD was conducted for both batch and CM granules using bottom sieve sizes of 1.0, 2.0, and 3.0 mm. Analysis showed that batch granules produced with a 1.0 mm sieve consisted of 39.88% fine powder (<100 μm), with no oversized granules (>850 μm, 0.00%). Under identical conditions, continuous process granules yielded 36.56 ± 4.75% fines and 0.03 ± 0.03% oversized granules, indicating a relative suppression of fine powder generation compared to the batch process. Within the continuous process, as the sieve size increased from 2.0 mm to 3.0 mm, the fine powder fraction decreased to 27.97 ± 4.09% and 29.08 ± 10.81%, respectively, while a corresponding increase in oversized granules (3.44 ± 1.56% and 6.35 ± 7.96%) was observed (Figure 1).

Notably, an increase in twin-screw speed, a critical parameter of the continuous process, led to a concomitant rise in both granule size and density. This can be fundamentally explained by the scale-independent concept of specific mechanical energy (SME); as screw speed escalates, the net mechanical energy transferred per unit mass of the powder bed increases significantly. This heightened SME delivers intensive shear forces and compressive energy within the screw channels, promoting highly efficient inter-particulate bonding and liquid distribution, which ultimately induces the formation of denser, less porous granules [21]. Particularly, when comparing the DoE midpoint of the continuous process (500 rpm, 2.0 mm) to the conventional batch process (1.0 mm), the milled granules exhibited highly analogous fraction patterns in both fines (<100 μm) and medium-sized granules. This structural and dimensional similarity in the milled granule state provides a solid scientific rationale for the excellent in vitro dissolution comparability (*f*_2_ > 50) between the two disparate manufacturing platforms.

### 2.2. Comparative Evaluation of Physical Tablet Properties

To evaluate the impact of disparate manufacturing processes on the physical quality of the final tablets, the hardness of tablets produced via batch and CM processes was comparatively analyzed under identical formulation and compression conditions. The results showed that the mean hardness of tablets produced by the batch process was 3.0 ± 0.5 kp, while those produced through the continuous process also exhibited a stable hardness distribution within the range of 2.0–4.0 kp. These data demonstrate that there were no statistically significant differences in mechanical strength between the two manufacturing methods. Consequently, this suggests that the continuous manufacturing process possesses sufficient process suitability to consistently produce tablets with physical properties equivalent to those of the conventional batch process.

A friability test was conducted to compare the mechanical strength and physical stability of tablets produced via batch and CM processes. The results revealed that tablets manufactured using the HSM-based batch process exhibited an average friability of 0.19%, demonstrating excellent physical resistance. Meanwhile, tablets from the TSG-based continuous process recorded an average friability of 0.51%. Although the continuous process tablets showed a relatively higher friability compared to those from the batch process, indicating a slight difference in mechanical integrity, both results were confirmed to meet the quality control specification of less than 1.0%. Consequently, these findings validate that both manufacturing methods possess the process suitability required to consistently produce high-quality tablets.

In this study, a comparative evaluation was conducted to investigate the impact of disparate manufacturing processes on tablet disintegration. While the batch process tablets recorded a rapid mean disintegration time of 1.67 min, the CM tablets showed a mean of 3.83 min, exhibiting relatively delayed release characteristics (Table 4).

In particular, an analysis of the correlation between twin-screw speed and disintegration time revealed a significant trend where higher RPM led to prolonged disintegration times. From a mechanistic perspective, this phenomenon is intimately tied to the interaction between SME and RTD. Although higher screw speeds typically shorten the mean residence time (RTD shift) within the barrel, the drastically elevated SME dominates the granulation process, generating highly densified granules with a rigid internal matrix. Consequently, when compressed, these dense granules yield tablets with a highly consolidated structural network that strongly suppresses water penetration into the tablet matrix, acting as a primary factor in the delayed disintegration [22,23].

### 2.3. Comparative Evaluation of In Vitro Dissolution Profiles

In this study, the in vitro dissolution profiles of tablets produced via batch and CM processes were comparatively analyzed to elucidate the impact of manufacturing differences on drug release behavior and evaluate the quality equivalence of the final formulations. These media (pH 1.2 and pH 4.0) were selected to secure analytical discriminatory power rather than strictly biorelevant intestinal simulation. Amiodarone HCl is a weakly basic, Biopharmaceutics Classification System Class II drug with highly pH-dependent solubility. Because it becomes practically insoluble at standard intestinal pH (pH 6.8), which masks process-induced variations, the intermediate pH 4.0 buffer was strategically utilized as an effective discriminatory medium. The dissolution rate at pH 4.0 is highly sensitive to the tablet matrix properties, allowing precise *f*_2_ profiling to define the safe operating boundaries of the continuous system.

**Figure 2:**
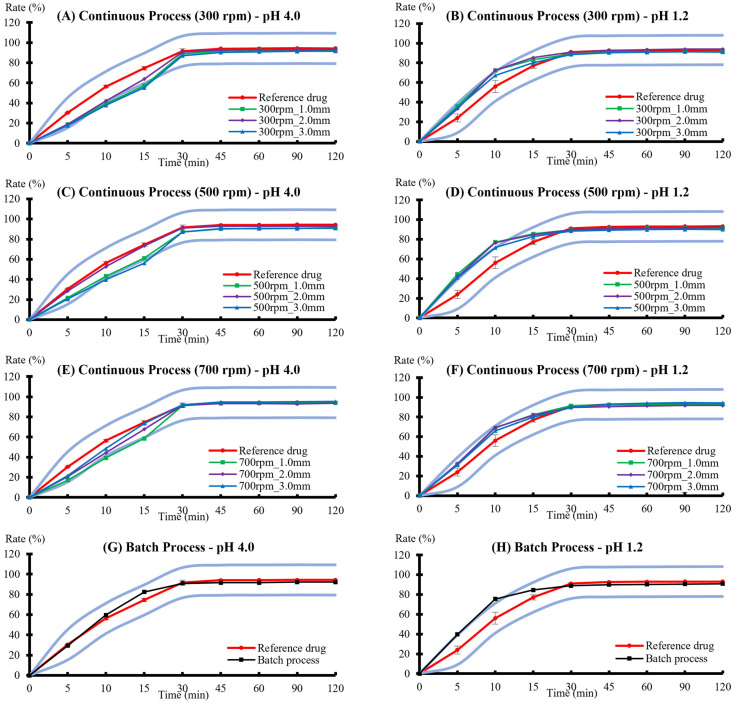
Comparative in vitro dissolution profiles of amiodarone HCl tablets manufactured via batch and continuous processes in pH 1.2 and pH 4.0 (1% Tween 80) media. (**A**) Continuous process (twin screw 300 rpm) in pH 4.0 medium. (**B**) Continuous process (twin screw 300 rpm) in pH 1.2 medium. (**C**) Continuous process (twin screw 500 rpm) in pH 4.0 medium. (**D**) Continuous process (twin screw 500 rpm) in pH 1.2 medium. (**E**) Continuous process (twin screw 700 rpm) in pH 4.0 medium. (**F**) Continuous process (twin screw 700 rpm) in pH 1.2 medium. (**G**) Batch process in pH 4.0 medium. (**H**) Batch process in pH 1.2 medium. The two light blue horizontal lines in each profile denote the upper and lower specification limits for target dissolution criteria.

The results indicated that the batch process tablets exhibited a rapid dissolution pattern exceeding target values under both gastric pH (mean 75.4 ± 0.1% at 10 min, 84.6 ± 1.3% at 15 min) and the intermediate pH 4.0 (mean 59.6 ± 1.7% at 10 min, 82.4 ± 0.3% at 15 min) conditions. Similarly, the continuous process tablets yielded results compliant with the established dissolution indices at gastric pH (72.1 ± 3.7% at 10 min, 83.3 ± 2.0% at 15 min) and the pH 4.0 medium (43.0 ± 4.9% at 10 min, 62.9 ± 7.1% at 15 min). Notably, it was confirmed that variations in key process parameters, such as twin-screw speed and milling size, did not induce significant fluctuations in the dissolution profiles (Figure 2). Finally, both formulations not only met the dissolution criteria for immediate-release products but also demonstrated statistical equivalence, as the similarity factor (*f*_2_) was calculated to be above 50. Consequently, it was concluded that quality equivalence between the two manufacturing methods was successfully established.

To systematically evaluate the risk of dissolution non-comparability across the different continuous process conditions, the *f*_2_ for each experimental run was calculated relative to the reference drug (Table 5). Although all experimental runs successfully satisfied the dissolution comparability criterion (*f*_2_ ≥ 50), the values varied depending on the process parameters. For instance, runs conducted under specific conditions exhibited *f*_2_ values closer to the borderline of 50, indicating a higher potential risk of non-comparability. Conversely, the optimized continuous process condition demonstrated a high *f*_2_ value, confirming robust dissolution equivalence to the reference drug. This comprehensive comparison of *f*_2_ values provides a clear scientific rationale for predicting the dissolution behavior and process risk within the continuous manufacturing platform.

### 2.4. Establishment of the Design Space

In this study, a CCD, a subclass of DoE, was employed to optimize the CPPs of the continuous process to ensure quality characteristics comparable to the conventional batch process. Based on a risk assessment, twin-screw speed (*X*_1_) and milling size (*X*_2_) were selected as the independent variables. The experimental ranges for these variables were established based on preliminary research data obtained from the batch process.

**Figure 3:**
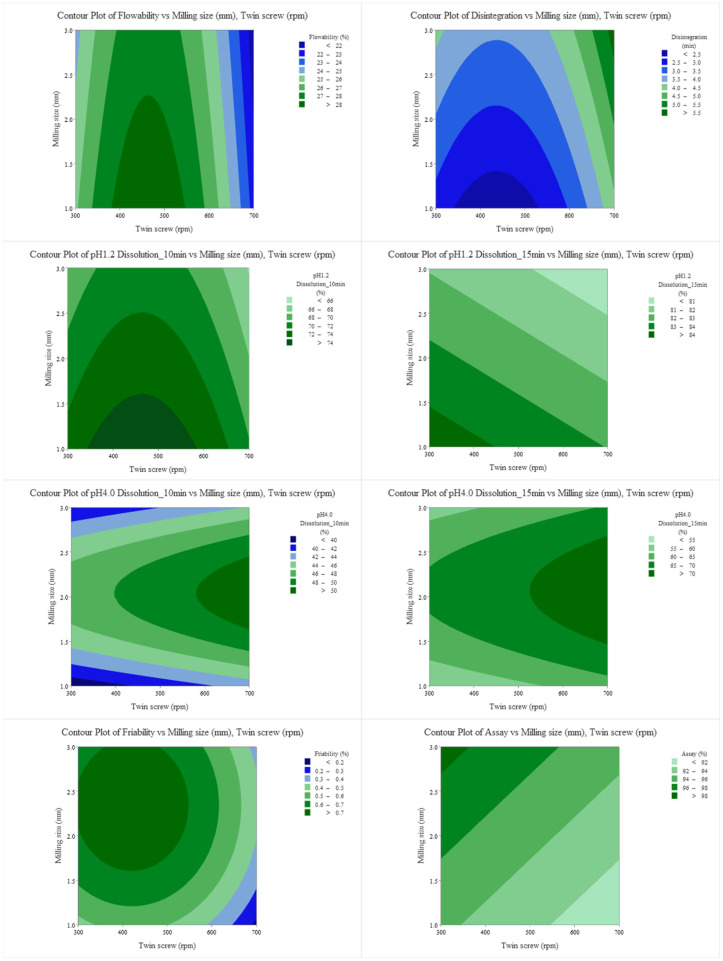
Contour plots showing the interaction effects of twin-screw speed and milling size on critical quality attributes.

Statistical analysis was conducted based on the results of the 13 experimental runs derived from the CCD. Contour plots were generated for each individual response to visually investigate the complex interactions between the variables (Table 6) (Figure 3).

Our DoE results confirmed that screw speed significantly impacts CQAs such as flowability, disintegration, and dissolution for this Amiodarone HCl tablet. However, it is important to note that while screw speed functions as a definitive CPP for this particular formulation matrix, its criticality may vary and might not be designated as a CPP for other formulations with different mechanical or physicochemical properties.

**Figure 4:**
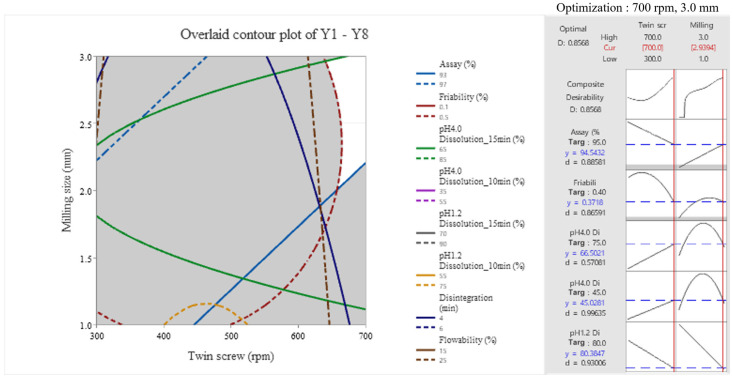
Multi-response optimization and determination of the continuous granulation DS (shaded region) with the finalized Optimal Operating Point (700 rpm, 3.0 mm).

In particular, to identify the optimal process region that simultaneously satisfies multiple CQAs—including flowability, disintegration, dissolution rates at various pH levels, friability, and assay—a DS was successfully established by overlaying the contour plots of each individual response. The theoretical optimal conditions calculated via the response optimizer showed an excellent desirability function value of 0.86 at a twin-screw speed of 700 rpm and a milling size of 2.9 mm. As illustrated by the shaded regions in Figure 4, a multidimensional Design Space was successfully justified where screw speed (approx. 630–700 rpm) and milling size (1.0–3.0 mm) can be varied simultaneously while satisfying all target criteria. Within this proven DS, 700 rpm and 3.0 mm were selected as the final single Optimal Operating Point (sweet spot) considering the actual equipment screen specifications and operational efficiency. At these finalized conditions, the desirability value was calculated to be 0.84, ensuring high reliability. Consequently, this provides a robust scientific basis for the stable production of high-quality tablets in the continuous process, equivalent to those produced by the conventional batch process.

### 2.5. Comparative Evaluation of Stability

In this study, a comparative stability study was conducted over 6 months under room temperature (RT) and accelerated storage (AC) conditions to evaluate the impact of different manufacturing processes on the stability of amiodarone HCl tablets. Assay (90.0–110.0%) and related substances (individual unknown impurity ≤ 0.2%, impurity D ≤ 0.5%, and total impurities ≤ 1.0%) were established as the CQAs for analysis (Table 7). At the 6-month time point, the mean assay values for the batch and continuous processes were recorded as 97.14% and 95.94%, respectively, strictly satisfying the established specifications for both methods. Furthermore, all impurity levels, including total related substances, remained consistently low within the specified limits, thereby demonstrating chemical stability.

In conclusion, the 6-month stability study successfully confirmed that the developed amiodarone HCl tablets provide equivalent stability to those from the batch process, even when produced via the continuous manufacturing process.

### 2.6. Comparison of Manufacturing Time and Costs Between Batch and Continuous Processes

As the final phase of this study, a comparative analysis of process efficiency and economic feasibility was conducted between the batch and CM processes. The total lead time from granulation to final tableting in the conventional batch process was approximately 10 h, comprising preparation and mixing (2 h), granulation (1 h), drying and quality control testing (5 h), milling and staging (1 h), and tableting (1 h). Analysis indicated that a significant portion of this duration was attributed to non-productive time, such as equipment re-setup between unit operations, material transfer, and particularly idle wait times for offline quality control approvals. Furthermore, the necessity for multiple skilled personnel at each stage and cumulative quality testing expenses were identified as primary drivers of high manufacturing costs.

In contrast, the continuous manufacturing process, where all unit operations are organically integrated into a single system, completed the entire sequence from setup to final product collection within 2 h. Regarding the cost structure, the high level of process automation minimized labor requirements, leading to a substantial reduction in personnel costs. In particular, the implementation of a real-time quality monitoring and assurance system utilizing PAT eliminated both the expenses and delays associated with offline quality testing, thereby maximizing economic efficiency.

To successfully scale up this platform for routine commercial manufacturing, an advanced PAT strategy enabling Real-Time Release Testing (RTRT) is required, such as embedding in-line NIR probes at the twin-screw discharge for moisture monitoring and at the final blending chute for real-time content uniformity verification. From a techno-economic perspective, however, implementing such a continuous platform demands higher initial capital and operating expenditures related to PAT systems. These financial burdens span three major phases: development costs (instrument procurement and chemometric modeling), operational costs (software licenses and specialized training), and maintenance costs (routine sensor re-validation and mandatory annual calibration). Despite these initial investment barriers and recurring maintenance costs, the long-term economic benefits—driven by a drastic reduction in batch rejection rates, minimized waste via automated diversion loops, and accelerated time-to-market—firmly justify the integration of a comprehensive PAT framework.

In conclusion, the continuous manufacturing process demonstrated an approximately 80% reduction in time and a 50% reduction in costs compared to the conventional batch process. These findings prove that CM offers a decisive advantage over batch processing in terms of both operational efficiency and economic feasibility, establishing its value as a next-generation standard for the production of high-value-added pharmaceuticals.

## 3. Materials and Method

### 3.1. Materials and Composition of Immediate-Release Formulation

Amiodarone HCl, the active pharmaceutical ingredient (API), was supplied by Zhejiang Hengkang Pharmaceutical Co., Ltd. (Taizhou, China). Additionally, Cordarone^®^ Tablets (200 mg amiodarone hydrochloride, Handok Inc., Seoul, Republic of Korea) were purchased from a local commercial pharmacy and utilized as the reference drug in the comparative in vitro dissolution studies (Figure 2), maintaining continuity with the target reference product analyzed in our prior batch-mode study [10]. The excipients used in the formulation included pre-gelatinized starch (Starch 1500; Colorcon Korea, Suwon, Republic of Korea), lactose monohydrate (Lactose monohydrate 200M; DFE Pharma, Goch, Germany), and polyvinylpyrrolidone K25 (Povidone K25; BASF, Ludwigshafen, Germany). Colloidal silicon dioxide (Aerosil 200; Evonik, Essen, Germany) and magnesium stearate (Nitika Pharmaceutical Specialties Pvt. Ltd., Nagpur, India) were utilized as a glidant and a lubricant, respectively. Acetonitrile and methanol for analytical purposes were purchased from Duksan Co., Ltd. (Ansan, Republic of Korea), and all other chemicals used were of analytical reagent grade.

Immediate-release Amiodarone HCl tablets were manufactured using both batch-wise HSM and continuous TSG. To ensure a valid comparison between the two processes, the same formulation optimized in a previous study was applied.

Wet granules were prepared by adding purified water to a homogeneous blend of the API and excipients (lactose monohydrate, pre-gelatinized starch, and povidone K25). The resulting granules were dried in an oven at 65 °C until the loss on drying (LOD), measured at 105 °C using a moisture analyzer (MB-120, OHAUS, Seoul, Republic of Korea), reached 2.0% or less. The dried granules were then milled using a hammer mill (PX-MFC 90D, KINEMATICA, Malters, Switzerland) equipped with a 1.0 mm screen.

Subsequently, colloidal silicon dioxide and magnesium stearate, sieved through 35-mesh and 25-mesh screens, respectively, were added for final blending. The final mixture was compressed into tablets with a target weight of 350 mg using a rotary tablet press (PR-LM08, PTK Co., Ltd., Gimpo, Republic of Korea). As an online PAT strategy, the real-time compression pressure monitoring system of the rotary tablet press was utilized. This continuous feedback mechanism allowed for precise adjustment of consolidation forces, ensuring that continuous tablets maintained rigid structural equivalence to the batch process. Consistent compression pressure was maintained across both manufacturing methods to rigorously evaluate quality equivalence.

### 3.2. Manufacturing Process Study

#### 3.2.1. Batch Wet Granulation Process

Batch-wise wet granulation was performed using a high-shear mixer (HSM, PM-C; PTK Co., Ltd., Gimpo, Republic of Korea). The process was divided into three distinct stages: mixing, kneading, and granulation. First, the pre-blended powder was loaded into the mixing bowl and mixed for 2 min with an impeller speed of 100 rpm and a chopper speed of 1500 rpm. Subsequently, the kneading stage was conducted for 3 min by adding purified water (the binding liquid) dropwise, while increasing the impeller speed to 250 rpm and maintaining the chopper speed at 1500 rpm. Following this, additional granulation was performed for 2 min under the same rotational conditions to finalize the granule formation. The resulting wet granules were dried in an oven at 65 °C until the LOD, measured at 105 °C using a moisture analyzer, reached 2.0% or less.

#### 3.2.2. Continuous Wet Granulation Process

**Figure 5:**
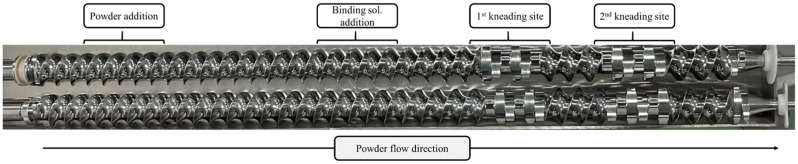
Screw configuration for the TSG process.

The continuous manufacturing process was conducted using a twin-screw granulator (TSG, ConsiGma™-1; GEA, Wommelgem, Belgium) (Figure 5). The equipment is a high-shear, co-rotating twin-screw granulator with a length-to-diameter (L/D) ratio of 20:1 and is operated without a die-plate. The screw configuration was specifically designed with a conveying section for forward powder transport and a mixing section to provide intensive shear force and facilitate the uniform mixing of the powder with purified water [24,25]. As key operational conditions, the barrel temperature was maintained at 25 °C and the powder feed rate was fixed at 13.0 kg/h. To maintain a strict formulation identity equivalent to the batch process, the liquid binding feed rate was continuously synchronized and fixed at a specific rate to keep the L/S ratio constant throughout all runs, ensuring that the final granule LOD consistently achieved ≤2.0%. Consequently, with the L/S ratio held as a controlled constant to preserve the composition matrix, the twin-screw rotation speed was systematically isolated and adjusted as the primary independent CPP according to the DoE.

The granulated material was automatically transferred to an integrated fluid bed dryer through a direct connection from the screw discharge. Drying was performed at an inlet air temperature of 65 °C with a drying air flow rate of 70 m^3^/h. The LOD of the final granules was precisely determined at 105 °C using a moisture analyzer, following the same protocol as the batch process.

### 3.3. Comparative Characterization of Granules

#### 3.3.1. Flowability

In this study, flowability was evaluated in accordance with USP <1174> guidelines by measuring bulk density (P_bulk_) and tapped density (P_tapped_), which were subsequently used to calculate CI and the Hausner Ratio (HR). Initially, an accurately weighed amount of granules was transferred into a 100 mL graduated cylinder to measure the initial bulk volume (P_bulk_), from which the bulk density was calculated. Subsequently, mechanical tapping was performed using a tapped density tester until a constant volume was achieved (at least 100 taps). The final tapped volume (P_tapped_) was then recorded to determine the tapped density. Each index was calculated using the following equations:

(1) $$Carr’s\;index\left(CI\right)=\;\frac{P_{Tapped}-P_{Bulk}}{P_{Tapped}}\times 100$$

(2) $$Hausner\;ratio\left(HR\right)=\frac{P_{Tapped}}{P_{Bulk}}$$

Regarding the criteria for flowability assessment, the granules were considered to have “good” flowability when the CI was 20% or less and the HR was less than 1.25, in accordance with the scale of flowability.

#### 3.3.2. Granule Content Assay

HPLC was conducted to assess the impact of rotational speed variations (impeller and screw speeds) on the content uniformity of amiodarone HCl within the granules produced via the two manufacturing processes. The analysis was performed using an Agilent 1260 Infinity II system (Agilent 1260 Infinity II, Agilent Technologies, Santa Clara, CA, USA) equipped with a ultraviolet-visible (UV-Vis) detector. Chromatographic separation was achieved using an Aegispak C18-L column (4.6 mm × 250 mm, 5.0 μm, YJ Biochrom, Seoul, Republic of Korea), with the column temperature maintained at 30 °C. The detection wavelength was set at 240 nm, the injection volume was 10 μL, and the flow rate of the mobile phase was 1.0 mL/min. The mobile phase was prepared by adding 3.0 mL of acetic acid and 20 mM triethylamine to 1 L of purified water, followed by precise pH adjustment to 3.0 using acetic acid. Finally, this aqueous buffer was mixed with acetonitrile in a volume ratio of 30:70 (*v*/*v*) and filtered for use in the analysis.

#### 3.3.3. Granule Particle Size Distribution

The PSD of the manufactured granules was evaluated by sieve analysis using a vibratory sieve shaker (AS-200; Retsch, Haan, Germany). A series of standard sieves with mesh sizes of 14, 20, 35, 40, 60, and 200 mesh were nested in descending order for the analysis. Approximately 10 g of the granule sample was accurately weighed and placed onto the uppermost sieve, followed by vibration for 10 min. After the completion of the sieving process, the mass of granules retained on each sieve was measured. Based on the obtained data, the weight percentage (% *w*/*w*) and cumulative weight percentage for each fraction were calculated to characterize the PSD of the granules.

### 3.4. Tablet Manufacturing Process Study

The Amiodarone HCl tablets were manufactured by compressing the granules prepared through the manufacturing process study described in Section 2.2. The tableting process was performed using a rotary tablet press (PR-LM08; PTK Co., Ltd., Gimpo, Republic of Korea) equipped with 10.5 mm standard round punches. To ensure uniformity in tablet quality, the target weight was established at 350 mg. The filling depth was fixed at 6.5 mm to facilitate consistent flow of the granules into the die and maintain a stable filling volume. Furthermore, to achieve optimal mechanical strength and physical stability, the compression force was precisely regulated at 3 kN. All manufactured tablets were collected and stored for subsequent quality characterization and evaluation.

### 3.5. Comparative Assessment Study of Tablets

The hardness of the manufactured amiodarone HCl tablets was measured using a tablet hardness tester (YD-II, LABOAO, Zhengzhou, China). Measurements were recorded in kp on tablets randomly sampled from each batch. To ensure physical stability without compromising disintegration and dissolution rates, the acceptance criteria for hardness were established at 4–7 kp. Consequently, only tablets within this specified range were utilized for subsequent studies and quality assessments.

Furthermore, the mechanical stability of the amiodarone HCl tablets was assessed. The test was conducted using a friability tester (CS-4, Mina Pharmaceutical Machinery Co., Ltd., Shanghai, China). For each experimental group, twelve tablets were randomly sampled, de-dusted, and their initial weight was precisely recorded. The tablets were then loaded into the drum and rotated for 100 revolutions at a speed of 25 rpm. Upon completion of the test, the tablets were de-dusted again to remove any loose powder, and their final weight was measured. The percentage of weight loss was subsequently calculated according to the following equation.

(3) $$Friability\;\left(\%\right)=\frac{W_{Initial}-W_{Final}}{W_{Initial}}\times 100\;(\%)$$

The acceptance criteria for quality were established such that a weight loss not exceeding 1.0% was defined as meeting specifications. This allowed for the validation of the mechanical integrity of the tablets following the transition of the manufacturing process.

Lastly, the disintegration time of the amiodarone HCl tablets was evaluated in accordance with USP <701> guidelines [26]. The test was conducted using a disintegration tester (BJ-2, Nanbei Instrument Ltd., Zhengzhou, China). Purified water, maintained at 37.0 ± 2.0 °C, was utilized as the immersion medium. Six tablets were placed in individual glass tubes within the basket assembly and tested simultaneously. The basket was oscillated vertically at a constant frequency. The time at which the tablets were completely broken down and passed through the wire mesh at the bottom of the tubes was recorded, and the mean disintegration time was calculated.

### 3.6. Dissolution Profiles

Dissolution tests of the amiodarone HCl tablets were conducted using USP Apparatus 2 (paddle method) in accordance with USP <711> Dissolution guidelines [27]. A 708-DS Dissolution Apparatus (Agilent Technologies, Santa Clara, CA, USA) was utilized for the study. Amiodarone HCl is categorized as a biopharmaceutics classification system Class II drug, characterized by low solubility and high permeability. Therefore, to ensure biorelevance and maintain sink conditions, 900 mL of pH 1.2 and pH 4.0 buffers supplemented with 1% (*w*/*v*) polysorbate 80 (Tween 80), a non-ionic surfactant, were employed as the dissolution media. The temperature was maintained at 37 ± 0.5 °C, and the paddle rotation speed was set at 75 rpm for a total duration of 120 min. Aliquots of 4 mL were withdrawn at designated time intervals (5, 10, 15, 30, 45, 60, 90, and 120 min) and immediately filtered through a 0.45 μm regenerated cellulose syringe filter for subsequent analysis.

The concentration of the analyte in the filtered dissolution samples was quantitatively analyzed using an HPLC system equipped with a UV-Vis detector. Separation was achieved using a Zorbax Eclipse Plus C18 column (4.6 mm × 150 mm, 5 μm, Agilent Technologies, Santa Clara, CA, USA). The analytical conditions were set as follows: detection wavelength of 240 nm, flow rate of 1.0 mL/min, injection volume of 10 μL, and column temperature of 40 °C. The mobile phase was prepared by mixing acetonitrile, methanol, and Solution A in a volume ratio of 42:38:20 (*v*/*v*/*v*). Solution A consisted of 1 L of purified water and 5 mL of triethylamine. The final mobile phase was adjusted to pH 4.0 using phosphoric acid.

The cumulative dissolution rate at each time point was calculated based on the measured concentrations using the following equation:

(4) $$\;Dissolution\;rate\;of\;a\;amiodarone\;hydrochloride\;\left(\%\right)=\;\frac{W_{S}\times A_{T}\times 9\times P}{A_{S}\times L}$$

where A_T_ and A_S_ are the peak areas of amiodarone HCl in the test and standard solutions, respectively; W_S_ is the weight of the amiodarone HCl standard; P is the purity of the standard (%); L is the labeled amount of amiodarone HCl per tablet; and 9 represents the dilution factor.

### 3.7. Design of Experiments

Statistical analysis was conducted using Minitab^®^ software (Version 22, Minitab Inc., State College, PA, USA). Twin-screw speed (*X*_1_) and milling size (*X*_2_) were selected as independent variables representing CPPs that influence the characteristics of granules and tablets. A face-centered CCD was employed to evaluate these factors across three levels, comprising a total of 13 experimental runs (4 factorial points, 4 axial points, and 5 center point replicates). The five replicates at the center point were performed to accurately estimate pure error and ensure model reproducibility. As dependent variables (*Y*), granule flowability, disintegration time, initial dissolution rates at pH 1.2 and pH 4.0 (at 10 and 15 min), friability, and assay were selected to analyze the main effects and interactions.

### 3.8. Stability Study

A stability study was conducted to evaluate the quality stability of amiodarone HCl tablets manufactured using both batch and continuous processes. The test samples were packaged and sealed in high-density polyethylene bottles and subsequently stored in stability chambers under the prescribed conditions. The storage conditions were established as RT conditions (25 ± 2 °C/60 ± 5% RH) and AC (40 ± 2 °C/75 ± 5% RH) for a total duration of 6 months. Sampling was performed at the initial time point and after 1, 3, and 6 months of storage. Each collected specimen was analyzed for appearance, assay, and related substances to evaluate any changes in pharmaceutical quality.

## 4. Conclusions

This study successfully demonstrated the transition of amiodarone HCl tablets from traditional HSM-based batch manufacturing to highly efficient TSG-based CM without any formulation changes. For scientific process design, twin-screw speed and milling size were identified as CPPs. By applying a CCD, the complex interactions between these variables were precisely analyzed, leading to the establishment of a robust DS region, from which the final optimal operating conditions were successfully derived. Analytical results indicated that the final tablets exhibited equivalence to the batch process in terms of CQAs, with no statistically significant differences observed. Notably, the equivalence of dissolution profiles served as a decisive indicator, underpinning the feasibility of the process transition without modifying the formulation. Regarding process efficiency, the CM process achieved a dramatic reduction in both total lead time (over 80%) and manufacturing costs (over 50%) by integrating unit operations and eliminating idle wait times, thereby proving its overwhelming economic advantage. In conclusion, this research proves that existing batch-based formulations can be flexibly transitioned to continuous manufacturing systems without compromising physicochemical quality, providing a vital technical foundation for the broader adoption of continuous processes in the pharmaceutical industry.

## Figures and Tables

**Table 1:** Established quality evaluation criteria and data comparison matrix for platform transition.

Quality Category	Specific Parameter	Target Criteria	Role in Current Study	Comparison Strategy
Mandatory (Critical release criteria)	Assay (%)	90.0–110.0%	Strict compliance required for product release. Direct indicators of therapeutic equivalence.	Individual Runs: Compared vs. Reference Drug to assess process risk boundaries. Optimized Sweet Spot: Compared vs. Batch and Reference Drug for final validation.
	Content uniformity	USP Compliant		
	Friability (%)	≤1.0%		
	Disintegration	USP Compliant		
	Dissolution (*f*_2_)	*f*_2_ ≥ 50		
Informative (Process indicators)	Flowability	Process suitability	Used as process performance indicators to evaluate granulation and milling efficiency.	Monitored across all DoE runs to map out the process Design Space.
	Particle size distribution	Trend monitoring		

**Table 2:** Comparison of flow properties of granules produced by batch and continuous processes.

Process	Continuous Process									Batch Process
Twin Screw (rpm)	300			500			700			
Milling Size (mm)	1.0 mm	2.0 mm	3.0 mm	1.0 mm	2.0 mm	3.0 mm	1.0 mm	2.0 mm	3.0 mm	1.0 mm
Bulk density (g/mL)	0.6583	0.7500	0.6728	0.6315	0.7000	0.6343	0.6985	0.7125	0.6512	0.6500
Tapped density (g/mL)	0.9307	0.9375	0.9122	0.8710	0.9333	0.8748	0.8950	0.9194	0.8288	0.7200
Carr’s Index (%)	29.3	20.0	26.3	27.5	25.0	27.5	22.0	22.5	21.4	9.7
Hausner’s Ratio	1.41	1.25	1.36	1.38	1.33	1.38	1.28	1.29	1.27	1.11
Flow character	Poor	Fair	Poor	Poor	Passable	Poor	Passable	Passable	Passable	Excellent

**Table 3:** Content uniformity results of granules from batch and continuous processes.

Process	Continuous Process									Batch Process
Twin Screw (rpm)	300			500			700			
Milling Size (mm)	1.0 mm	2.0 mm	3.0 mm	1.0 mm	2.0 mm	3.0 mm	1.0 mm	2.0 mm	3.0 mm	1.0 mm
Content Uniformity (%)	93.7	95.2	94.7	92.1	94.3	93.1	95.1	95.2	95.1	96.2
	92.8	95.6	93.8	92.3	94.0	93.6	94.5	94.9	95.5	98.0
	92.5	95.8	95.0	91.7	94.4	93.0	96.0	93.7	97.0	96.0
Average (%)	93.0 ± 0.6	95.5 ± 0.3	94.5 ± 0.6	92.0 ± 0.3	94.3 ± 0.2	93.2 ± 0.3	95.2 ± 0.8	94.6 ± 0.8	95.8 ± 1.0	96.8 ± 1.1

**Table 4:** Comparative analysis of physical properties for tablets produced by batch and continuous processes.

Process	Continuous Process									Batch Process
Twin Screw (rpm)	300			500			700			
Milling Size (mm)	1.0 mm	2.0 mm	3.0 mm	1.0 mm	2.0 mm	3.0 mm	1.0 mm	2.0 mm	3.0 mm	1.0 mm
Hardness (kp)	3.9	2.7	1.8	3.8	2.9	1.8	4.0	3.1	2.2	3.0
Friability (%)	0.38	0.67	0.76	0.57	0.66	0.66	0.19	0.47	0.28	0.19
Disintegration (min)	3.20	2.95	4.23	2.13	2.80	4.03	4.42	5.13	5.58	1.67

**Table 5:** Calculated dissolution similarity factor (*f*_2_) of each experimental run compared to the reference drug.

No.	Twin Screw (rpm)	Milling Size (mm)	pH 4.0 *f*_2_ Value	pH 1.2 *f*_2_ Value	Risk Assessment
1	300	1.0	51.3	58.4	Moderate Risk
2	300	2.0	58.2	57.6	Low Risk
3	300	3.0	50.2	65.6	High Risk
4	500	1.0	55.2	53.1	Moderate Risk
5	500	2.0	84.4	53.8	Moderate Risk
6	500	3.0	50.1	58.3	High Risk
7	700	1.0	52.5	68.3	Moderate Risk
8	700	2.0	63.6	55.5	Moderate Risk
9	700	3.0	74.7	61.3	Extremely Safe
11	Batch process	1.0	70.9	54.4	Moderate Risk

**Table 6:** Central composite design matrix: Independent variables (*X*_1_, *X*_2_) and their experimental responses (*Y*_1_–*Y*_8_).

Run Order	Independent Variables		Dependent Variables							
	Twin Screw (rpm)	Milling Size (mm)	Flowability (%)	Disintegration (min)	pH 1.2 Dissolution 10 min (%)	pH 1.2 Dissolution 15 min (%)	pH 4.0 Dissolution 10 min (%)	pH 4.0 Dissolution 15 min (%)	Friability (%)	Assay (%)
	*X*_1_	*X*_2_	*Y*_1_	*Y*_2_	*Y*_3_	*Y*_4_	*Y*_5_	*Y*_6_	*Y*_7_	*Y*_8_
1	300	1.0	29.3	3.20	72.3	83.1	39.4	57.2	0.38	94.3
2	700	1.0	22.0	4.42	68.9	82.2	39.3	58.5	0.19	91.9
3	300	3.0	26.3	4.23	67.3	80.3	37.7	55.2	0.76	101.4
4	700	3.0	21.4	5.58	66.0	79.8	48.6	73.5	0.28	95.2
5	300	2.0	20.0	2.95	72.5	85.3	42.1	63.9	0.67	95.5
6	700	2.0	22.5	5.13	69.3	81.8	44.4	67.7	0.47	92.2
7	500	1.0	27.5	2.13	77.0	85.5	43.2	61.0	0.57	91.0
8	500	3.0	27.5	4.03	71.6	82.7	39.6	56.1	0.66	93.2
9	500	2.0	27.5	2.80	69.2	79.9	49.4	68.2	0.66	92.4
10	500	2.0	28.8	3.02	71.5	82.2	52.6	71.4	0.76	94.0
11	500	2.0	28.7	3.15	76.5	84.6	52.7	73.3	0.76	97.6
12	500	2.0	27.3	2.98	72.4	82.2	51.6	71.0	0.66	94.7
13	500	2.0	28.8	3.05	72.9	82.3	51.0	70.8	0.76	95.8

**Table 7:** Comparative stability results of batch and continuous manufacturing processes.

Amiodarone Hydrochloride					
Sample	Storage Condition *	Time	Assay (%)	Impurity D (%)	Total Impurity (%)
Continuous tablet	-	Initial	94.4	0.06	0.15
	RT	1 M	95.5	0.06	0.14
		3 M	98.6	0.07	0.20
		6 M	98.6	0.07	0.21
	AC	1 M	93.4	0.20	0.28
		3 M	94.9	0.32	0.45
		6 M	97.9	0.45	0.58
Batch tablet	-	Initial	97.7	0.05	0.14
	RT	1 M	94.2	0.05	0.12
		3 M	102.4	0.06	0.18
		6 M	97.7	0.07	0.20
	AC	1 M	94.6	0.20	0.26
		3 M	95.4	0.35	0.46
		6 M	98.0	0.46	0.59

* RT: Long-term storage conditions (25 ± 2 °C/60 ± 5% relative humidity (RH)), AC: Accelerated conditions (40 ± 2 °C/75 ± 5% RH).

## Data Availability

The original contributions presented in this study are included in the article. Further enquiries can be directed to the corresponding author.

## Abbreviations

The following abbreviations are used in this manuscript:

CM	continuous manufacturing
CPPs	critical Process Parameters
PAT	process analytical technology
CMAs	critical material attributes
CQAs	critical quality attributes
HSM	high shear mixer
TSG	Twin-screw granulation
RTD	residence time distribution
PSD	particle size distribution
DS	design space
HCl	hydrochloride
DoE	Design of Experiment
CCD	central composite design
USP	United States Pharmacopeia
CI	Carr’s Index
HPLC	High-performance liquid chromatography
SME	specific mechanical energy
RT	room temperature
AC	accelerated storage
RH	relative humidity
API	active pharmaceutical ingredient
LOD	loss on drying
HR	Hausner Ratio
UV-Vis	ultraviolet-visible
Tween 80	polysorbate 80
